# Case Report: Onset of Type 1 Diabetes Mellitus in a Patient With Ulcerative Colitis and Sjogren’s Syndrome Under Euthyroid Hashimoto’s Thyroiditis

**DOI:** 10.3389/fendo.2022.836102

**Published:** 2022-03-17

**Authors:** Kaio Takahashi, Takatoshi Anno, Akio Matsuda, Yukiko Kimura, Fumiko Kawasaki, Kohei Kaku, Koichi Tomoda, Hirofumi Kawamoto, Hideaki Kaneto

**Affiliations:** ^1^ Department of General Internal Medicine 1, Kawasaki Medical School, Okayama, Japan; ^2^ Department of General Internal Medicine 2, Kawasaki Medical School, Okayama, Japan; ^3^ Department of Diabetes, Endocrinology and Metabolism, Kawasaki Medical School, Kurashiki, Japan

**Keywords:** type 1 diabetes mellitus, autoimmune polyglandular syndrome (APS), ulcerative colitis, Sjogren’s syndrome, euthyroid Hashimoto’s thyroiditis

## Abstract

Type 1 diabetes mellitus (T1DM) is often complicated with some other autoimmune disorders. The complication of various autoimmune disorders is known as autoimmune polyglandular syndrome (APS). Once autoimmune thyroid disease develops, various autoimmune diseases can also occur. Such phenomena are classified as APS types 3A to 3D. In this report, we show the onset of T1DM in a patient with ulcerative colitis (UC) and Sjogren’s syndrome. The most important and interesting point in this case is that, if we did not check her thyroid-associated antibodies, we could not have diagnosed her as APS. From the data of this case, we assumed that the patient suffered from APS type 3A, 3B, and 3D variants. This case pointed out very clearly the importance of testing for thyroid-associated antibodies under various autoimmune disease conditions even if the thyroid hormone levels are euthyroid. Moreover, based on the strong linkage between inflammatory bowel disease and T1DM and the compatibility with both T1DM and APS type 3, we think it is possible that Hashimoto’s disease is present under complicated conditions together with UC and T1DM. It would be important to repeatedly check for thyroid-associated antibodies even in euthyroid patients, especially under various autoimmune disease conditions.

## Introduction

Autoimmune polyglandular syndrome (APS) differs in their component diseases, which are a group of syndromes comprising a combination of endocrine and other autoimmune diseases, and in the immunological features of their pathogenesis (1, 2). Four major entities are recognized—APS types 1–4 and APS type 3—with type 3 being the most common type (3). While the coexistence of autoimmune Addison’s disease defines classification into APS types 1, 2, and 4, APS type 3 does not include adrenal failure. Type 1 diabetes mellitus (T1DM) is often complicated with some other autoimmune disorders. The complication of various autoimmune disorders is known as APS (1). APS type 3A consists of T1DM and autoimmune thyroid diseases such as Basedow’s disease and Hashimoto’s thyroiditis. However, once autoimmune thyroid disease develops, various autoimmune diseases can also occur. Such phenomena are classified as APS types 3A to 3D (2). It should be noted that, differently from the initial observations where APS constituted only of clinical autoimmune diseases, presently, APS can also be diagnosed in the presence of one or more clinical and one or more subclinical or potential (e.g., only positive for autoantibodies) autoimmune diseases (4).

Ulcerative colitis is an inflammatory bowel disease (IBD) that causes inflammation and ulcers in the digestive tract, and one possible cause considered is an immune system malfunction, although the exact cause of ulcerative colitis (UC) remains unknown. Interestingly, several studies have reported a strong linkage between IBD and T1DM. It has been suggested that these two diseases share similar immune-mediated pathogenesis, which indicates a potential epidemiologic association (5–7).

In this report, we show the onset of T1DM in a patient with UC and Sjogren’s syndrome. Interestingly, the patient’s antibodies for Hashimoto’s thyroiditis [thyroid peroxidase antibody (TPOAb) and thyroglobulin antibody (TgAb)] were of high titers, although her thyroid hormone levels were euthyroid. From these results, we assumed that she suffered from APS type 3A, 3B, and 3D variants.

## Case Description

A 53-year-old Japanese woman was referred to our hospital for hyperglycemia, where she underwent lower gastrointestinal endoscopy for UC. Her medical history included UC at age 18, and she had repeated remissions and exacerbations. She was taking 1,200–3,600 mg/day of 5-aminosalicylic acid, when necessary. In addition, she was diagnosed with Sjogren’s syndrome at age 51. Schirmer’s test did not show deficient tear production, which had wetting of 6 mm. Since she did not have sicca symptoms, she was not given medication. Her height, body weight, and body mass index were 154.1 cm, 53.5 kg, and 22.5 kg/m^2^, respectively. Her vital signs were as follows: temperature, 36.4°C; blood pressure, 134/84 mmHg; heart rate, 67 bpm; and oxygen saturation, 97% (room air). As shown in **Table 1**, the patient’s diabetes-associated data were as follows: plasma glucose (PG), 385 mg/dl; glycated hemoglobin, 11.3%; glycoalbumin, 31.8%; plasma insulin (PI), 1.2 μU/ml; and C-peptide immunoreactivity (CPR), 3.7 ng/ml. Ketone body was not detected. In addition, autoimmune markers of diabetes mellitus were as follows: anti-glutamic acid decarboxylase (GAD) antibody, 16,878.7 U/ml; anti-insulinoma-associated antigen 2 antibody, negative; anti-islet cell antibody, negative; anti-zinc transporter 8 antibody, negative; and anti-insulin antibody, negative. Since her anti-GAD antibody was positive and her insulin secretion was markedly suppressed, this patient was diagnosed with T1DM and insulin therapy was started. Anti-nuclear antibody (ANA), anti-SS-A/Ro antibody, anti-SS-B/La antibody, and rheumatoid factor (RF) were all positive [ANA, 105(+); anti-SS-A/Ro, ≥256 U/ml; anti-SS-B/La, 16.3 U/ml; RF, 165 IU/ml]. Renal function, liver function, and other endocrine hormone levels were within the normal range. Since she was diagnosed with UC, Sjogren’s syndrome, and T1DM at that time, we examined other thyroid-associated antibodies even though she was euthyroid [thyroid-stimulating hormone (TSH), 1.649 μIU/ml; free triiodothyronine (FT3), 3.18 pg/ml; and free thyroxine (FT4), 0.92 ng/dl]. TPOAb and TgAb were elevated to high titers (≥600 and 277.5 IU/ml, respectively) (**Table 2**). Ultrasound examination of the thyroid revealed that the thyroid gland was not hypervascular and was slightly low and hetero-echo, although it was not enlarged. As she had various autoimmune disorders, such as euthyroid Hashimoto’s thyroiditis, UC, Sjogren’s syndrome, and T1DM, she was diagnosed with APS type 3A, 3B, and 3D variants. The results of human leukocyte antigen (HLA) DNA typing were as follows: *DRB1* 04:05, 12:02; *DQB1* 03:01, 04:01. These were also compatible with T1DM and APS type 3 (7). Hyperglycemia was gradually resolved with insulin therapy (6 U/day of insulin aspart and 3 U/day of insulin degludec).

**Table 1:** Laboratory data at the onset of type 1 diabetes mellitus in this patient.

Variable	Result	Reference range	Variable	Result	Reference range
**Peripheral blood**			**Diabetes and lipid marker**		
White blood cells (/μl)	3,340	3,300–8,600	Plasma glucose (mg/dl)	385	
Red blood cells (×10^4^/μl)	446	386–492	plasma insulin (μU/ml)	1.2	1.84–12.2
Hemoglobin (g/dl)	12.9	11.6–14.8	CPR (ng/ml)	3.70	0.61–2.09
Hematocrit (%)	38.0	35.1–44.4	Hemoglobin A1c (%)	11.3	4.6–6.2
Platelets (×10^4^/μl)	22.6	15.8–34.8	Glycoalbumin (%)	31.8	12.4–16.3
**Blood biochemistry**			Total cholesterol (mg/dl)	159	142–248
Total protein (g/dl)	7.8	6.6–8.1	LDL cholesterol (mg/dl)	40	65–139
Albumin (g/dl)	4.3	4.1–5.1	HDL cholesterol (mg/dl)	55	40–90
Globulin (g/dl)	3.5	2.2–3.4	Triglyceride (mg/dl)	87	40–149
Total bilirubin (mg/dl)	0.9	0.4–1.5	**Endocrine marker**		
Direct bilirubin (%)	16	30–52	TSH (μU/ml)	1.649	0.400–6.000
AST (U/L)	22	13–30	Free triiodothyronine (pg/ml)	3.18	2.5–4.20
ALT (U/L)	14	7–23	Free thyroxine (ng/dl)	0.92	0.80–1.60
LDH (U/L)	163	124–222	ACTH (pg/ml)	31.4	7.2–63.3
ALP (U/L)	288	106–322	Cortisol (μg/dl)	15.3	4.5–21.1
γ-GTP (U/L)	22	9–32	DHEA-S (μg/dl)	121	13–154
BUN (mg/dl)	16	8–20	Aldosterone (pg/ml)	174	4.0–82.1
Creatinine (mg/dl)	0.69	0.64–0.79	Renin activity (ng ml^−1^ h^−1^)	0.7	0.2–3.9
Uric acid (mg/dl)	4.3	2.6–5.5	**Urinary test**		
Amylase (μg/dl)	129	44–132	Urinary pH	7.0	5.0–7.5
CRP (mg/dl)	0.06	<0.14	Urinary protein	–	–
Sodium (mmol/L)	142	138–145	Urinary sugar	3+	–
Potassium (mmol/L)	3.6	3.6–4.8	Urinary ketone body	1+	–
Chloride (mmol/L)	106	101–108	Urinary bilirubin	–	–
IP (mg/dl)	3.9	2.7–4.6	Urinary blood	–	–
Calcium (mg/dl)	9.0	8.8–10.1			
Magnesium (mg/dl)	2.0	1.9–2.6			

AST, aspartate aminotransferase; ALT, alanine aminotransferase; LDH, lactate dehydrogenase; ALP, alkaline phosphatase; γ-GTP, γ-glutamyl transpeptidase; BUN, blood urea nitrogen; CRP, C-reactive protein; IP, inorganic phosphorus; CPR, C-peptide immunoreactivity; LDL, low-density lipoprotein; HDL, high-density lipoprotein; TSH, thyroid-stimulating hormone; ACTH, adrenocorticotropic hormone; DHEA-S, dehydroepiandrosterone sulfate.

**Table 2:** Disease-specific antibodies and HLA DNA typing in this subject.

Variable	Result	Reference range
Anti-glutamic acid decarboxylase Ab (U/ml)	1,6878.7	<5.0
Anti-insulinoma-associated protein 2 Ab (U/ml)	Negative	0–0.3
Ant-islet cell Ab (JDF unit)	Negative	<1.25
Anti-zinc transporter 8 Ab (U/ml)	Negative	<15.0
Anti-insulin Ab (nU/ml)	<125.0	<125.0
Anti-nuclear Ab	105 (+)	<20.0
Rheumatoid factor (U/ml)	165	0–15
Anti-ds-DNA Ab (IU/ml)	<10	0–12
Anti-ribonucleoprotein Ab (U/ml)	<2.0	<10.0
Anti-Smith Ab (U/ml)	3.0	<10.0
Anti-Scl-70 Ab (U/ml)	1.8	<10.0
Anti-centromere Ab (index)	<5.0 (−)	<10.0
Anti-Jo-1 Ab (U/ml)	2.6	<10.0
Lupus anticoagulant	1.07	<1.3
Anti-cardiolipin immunoglobulin G Ab (U/ml)	≤1.2	<3.5
Anti-cardiolipin β2-glycoprotein I Ab (U/ml)	≤8	<10
Anti-SS-A/Ro Ab (U/ml)	≥256	Negative
Anti-SS-B/La Ab (U/ml)	16.3	Negative
Anti-cyclic citrullinated peptide Ab (U/ml)	<0.5	<4.5
Matrix metalloproteinase-3 (ng/ml)	50.3	17.3–59.7
Myeloperoxidase–anti-neutrophil cytoplasmic antibody (U/ml)	<1.0	<3.5
Proteinase 3-antineutrophil cytoplasmic antibody (U/ml)	1.3	<3.5
Anti-aminoacyl-tRNA synthetase Ab	6.3 negative	<25.0
Antimitochondrial M2 antibody	<1.5 negative	<7.0
Anti-thyrotropin receptor Ab (IU/L)	<1.0	<1.0
Anti-thyroid stimulating Ab (%)	110	0–120
Anti-thyroid peroxidase Ab (IU/ml)	≥600	<16.0
Anti-thyroglobulin Ab (IU/ml)	277.5	<28.0
HLA DNA typing	*DRB1* 04:05, 12:02 *DQB1* 03:01, 04:01	

HLA, human leukocyte antigen; Ab, antibody; JDF, Juvenile Diabetes Foundation.

**Figure 1:**
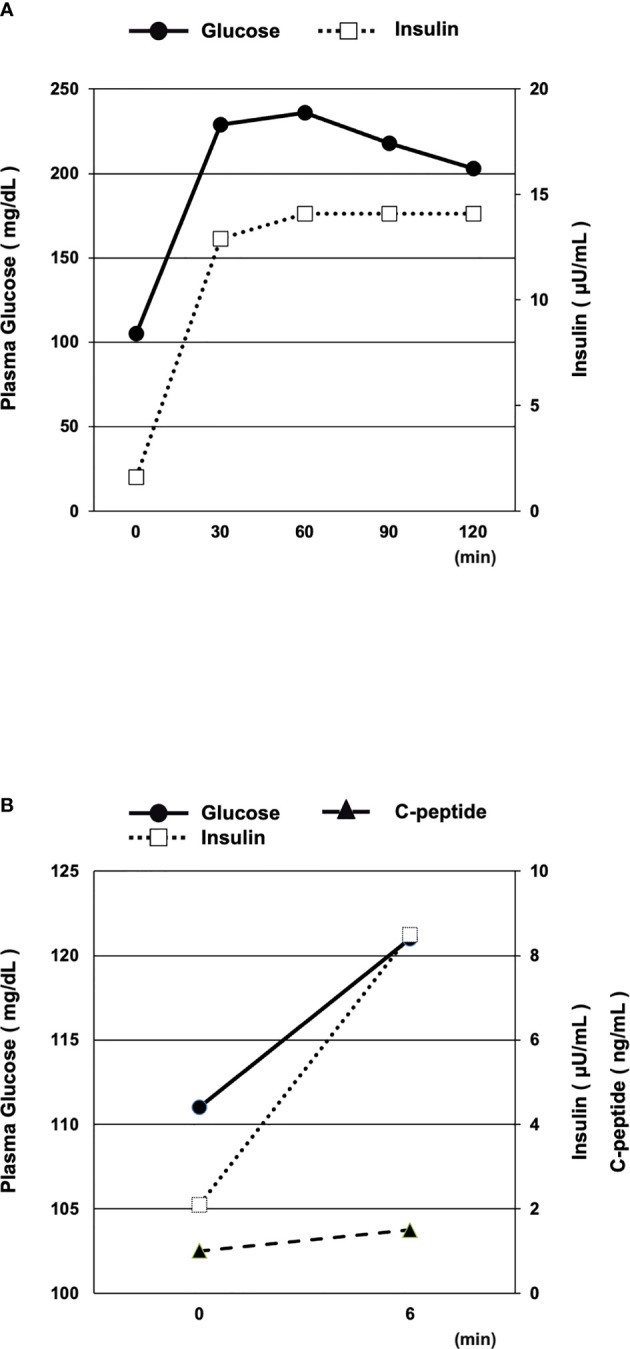
**(A, B)** Oral glucose tolerance test (OGTT, 75 g) **(A)** and glucagon stimulation test (GST) **(B)** after reduction of glucose toxicity. Both the 75 g OGTT and GST results revealed that the patient’s insulin secretory capacity was decreased, although not exhausted.

We examined her insulin secretion level with 75 g oral glucose tolerance test (OGTT) and glucagon stimulation test (GST) after reduction of glucose toxicity. The OGTT (75 g) results were as follows (**Figure 1A**): fasting, PG = 105 mg/dl, PI = 1.6 μU/ml; 30 min, PG = 229 mg/dl, PI 12.9 μU/ml; 60 min, PG = 236 mg/dl, PI = 14.1 μU/ml; 90 min, PG = 218 mg/dl, PI = 14.1 μU/ml; 120 min, PG = 203 mg/dl, PI = 14.1 μU/ml. We calculated the change in C-peptide immunoreactivity (ΔCPR) by subtracting fasting CPR (PG = 111 mg/dl, PI = 2.1 μU/ml, CPR = 1.0 ng/ml) from the CPR result at 6 min after injection of 1 mg glucagon (PG = 121 mg/dl, PI = 8.5 μU/ml, CPR = 1.5 ng/ml). The ΔCPR was 0.5 ng/ml (**Figure 1B**). Both 75 g OGTT and GST revealed that her insulin secretory capacity was decreased, although it was not exhausted.

## Discussion

Herein, we reported a case of onset of T1DM in a patient with UC complicated with Sjogren’s syndrome. Interestingly, her thyroid-associated antibodies (TPOAb and TgAb) showed high titers, even though her thyroid hormone levels were euthyroid. This result meant that, if we did not examine the thyroid-associated antibodies, this patient (with T1DM + UC + Sjogren’s syndrome) would have been diagnosed as APS type 4.

In 1980, Neufeld and Blizzard organized and classified these clinical clusters into four main types defined as APS, which are summarized in **Table 3** (8). The coexistence of autoimmune Addison’s disease is defined as APS types 1, 2, and 4. In addition, the prevalence rates of clinical autoimmune diseases in a cumulative population with autoimmune Addison’s disease were: IBD, 2.4%; Sjogren’s syndrome, 2.4%; T1DM, 1.2%–20.4%; and Hashimoto’s thyroiditis, 3.7%–32% (2). On the other hand, the characteristics of APS type 3 are shown in **Table 4**. This condition is characterized by autoimmune thyroiditis along with other organ-specific autoimmune diseases. Our patient was diagnosed with the APS type 3A, 3B, and 3D variants together with euthyroid Hashimoto’s thyroiditis. Moreover, comparison of the prevalence rates between TPOAb and TgAb in healthy controls showed values of about 1.2%–27.8% and 1.2%–30%, respectively, in various places in different countries (9). Since her TPOAb and TgAb were of very high titers, she was diagnosed with euthyroid Hashimoto’s thyroiditis, even though she suffered from potential Hashimoto’s thyroiditis.

**Table 3:** Classification of autoimmune polyglandular syndrome (APS) according to Neufeld and Blizzard (2, 4, 8).

APS type 1	Chronic candidiasis, chronic hypoparathyroidism, autoimmune Addison’s disease (at least two present)
APS type 2	Autoimmune Addison’s disease + autoimmune thyroid diseases and/or type 1 diabetes mellitus (Addison’s disease must always be present)
APS type 3	Thyroid autoimmune diseases + other autoimmune diseases (excluding autoimmune Addison’s disease, hypoparathyroidism, and chronic candidiasis)
APS type 4	Two or more organ-specific autoimmune diseases (which do not fall into type 1, 2, or 3)

**Table 4:** Characteristics of autoimmune polyglandular syndrome (APS) type 3 (2, 4).

Autoimmune thyroid diseases			
Hashimoto’s thyroiditisAsymptomatic autoimmune thyroiditis	Graves’ disease		Idiopathic myxedema Endocrine ophthalmopathy
+	+	+	+
Type 1 diabetes mellitus Hirata’s disease Lymphocytic hypophysitis POF	Chronic atrophic gastritis Pernicious anemia Celiac disease Chronic inflammatory bowel diseases Autoimmune hepatitis Primary biliary cirrhosis	Vitiligo Alopecia Myasthenia gravis Stiff-man syndrome Multiple sclerosis	SLE or DLE Mixed connective tissue disease Rheumatoid arthritis Seronegative arthritis Systemic sclerosis Sjogren’s syndrome Werlhof syndrome Antiphospholipid syndrome Vasculitis
3A	3B	3C	3D

POF, premature ovarian failure; SLE, systemic lupus erythematosus; DLE, discoid lupus erythematosus.

UC is an IBD and a chronic autoimmune condition affecting the gastrointestinal tract as the pathogenesis mechanism (10), although various genetic and environmental factors have been implicated in UC susceptibility (11). It is therefore important to understand the relationship between IBD and T1DM in clinical practice. Genetic research revealed a linkage between IBD and T1DM and identified that both share risk variants at 20 loci, which is 10 times higher than that expected by chance (12). Most of the overlap genes were related to inflammatory response, which strongly indicated that the two diseases share similar immune-mediated pathogenesis (12, 13), although the mechanisms of the immunological pathogenesis of the linkage between IBD and T1DM are unknown. On the other hand, some clinical research studies and meta-analyses have identified the association between IBD and T1DM (13–15). Although it is unclear whether UC is a chronic autoimmune condition, Betterle et al. recently included UC, Crohn’s disease, celiac disease, and autoimmune pancreatitis as gastrointestinal autoimmune diseases in the classification of APS type 3B (4). Considering that thyroid autoimmune disease (in clinical, subclinical, or potential form) can be the most frequent disease associated with any other autoimmune diseases, we examined TPOAb and TgAb despite the presence of a normal thyroid function in a patient with multiple autoimmune diseases. On the basis of this investigation, we identified a potential combination of multiple variants of APS type 3. In this case, she suffered from APS type 3A, 3B, and 3D variants because she experienced the onset of T1DM and had thyroid autoantibodies.

The most important and interesting point in this case is that, if we did not check her thyroid-associated antibodies, we could not have diagnosed her with APS. Generally, when the thyroid hormone levels are euthyroid, thyroid-associated antibodies are not measured. However, our patient suffered from UC and Sjogren’s syndrome during the onset of T1DM, suggesting that she had various autoimmune disease conditions. In addition, her HLA DNA typing results showed *DRB1* 04:05 and *DQB1* 04:01, which were also compatible with both T1DM and APS type 3 (16). This case pointed out very clearly the importance of measuring thyroid-associated antibodies under various autoimmune disease conditions even if the thyroid hormone levels are euthyroid. Moreover, based on the strong linkage between IBD and T1DM and the compatibility with both T1DM and APS type 3, we think it is possible that Hashimoto’s disease is present under complicated conditions together with UC and T1DM.

## Conclusion

Taken together, it should be noted that a patient can suffer from APS type 3A, 3B, and 3D variants. In addition, if we did not check our patient’s thyroid associated antibodies, we could not have diagnosed her with the correct APS variation. Therefore, it is important that all clinicians who follow patients with one or more autoimmune diseases must examine the thyroid associated antibodies regardless of the presence or absence of a thyroid dysfunction, at least once (or, if possible, repeatedly as much as costs permit).

## Data Availability Statement

The original contributions presented in the study are included in the article/supplementary material. Further inquiries can be directed to the corresponding author.

## Ethics Statement

Written informed consent was obtained from the individual(s) for the publication of any potentially identifiable images or data included in this article. Informed consent was obtained from the patient for inclusion in the case report.

## Author Contributions

TA researched data and wrote the manuscript. KaT, AM, YK, and FK researched data contributed to the discussion. KK, KoT, HirK, and HidK reviewed the manuscript.

## Funding

The author(s) received no financial support for the research, authorship, and/or publication of this article.

## Conflict of Interest

The authors declare that the research was conducted in the absence of any commercial or financial relationships that could be construed as a potential conflict of interest.

## Publisher’s Note

All claims expressed in this article are solely those of the authors and do not necessarily represent those of their affiliated organizations, or those of the publisher, the editors and the reviewers. Any product that may be evaluated in this article, or claim that may be made by its manufacturer, is not guaranteed or endorsed by the publisher.

## References

[1] Michels AW Gottlieb PA. Autoimmune Polyglandular Syndromes. Nat Rev Endocrinol (2010) 6:270–7. doi: 10.1038/nrendo.2010.40 20309000

[2] Betterle C Dal Pra C Mantero F Zanchetta R. Autoimmune Adrenal Insufficiency and Autoimmune Polyendocrine Syndromes: Autoantibodies, Autoantigens, and Their Applicability in Diagnosis and Disease Prediction. Endocr Rev (2002) 23:327–64. doi: 10.1210/edrv.23.3.0466 12050123

[3] Huber A Menconi F Corathers S Jacobson EM Tomer Y. Joint Genetic Susceptibility to Type 1 Diabetes and Autoimmune Thyroiditis: From Epidemiology to Mechanisms. Endocr Rev (2008) 29:697–725. doi: 10.1210/er.2008-0015 18776148 PMC2583387

[4] Betterle C Sabbadin C Scaroni C Presotto F. Autoimmune Polyendocrine Syndromes (APS) or Multiple Autoimmune Syndromes (MAS). In: Colao A Jaffrain-Rea ML Beckers A, editors. Polyendocrine Disorders and Endocrine Neoplastic Syndromes 2021; Endocrinology. Cham: Springer. doi

[5] Wang K Baldassano R Zhang H Qu HQ Imielinski M Kugathasan S. Comparative Genetic Analysis of Inflammatory Bowel Disease and Type 1 Diabetes Implicates Multiple Loci With Opposite Effects. Hum Mol Genet (2010) 19:2059–67. doi: 10.1093/hmg/ddq078 PMC2860894 20176734

[6] Gjymishka A Coman RM Brusko TM Glover SC. Influence of Host Immunoregulatory Genes, ER Stress and Gut Microbiota on the Shared Pathogenesis of Inflammatory Bowel Disease and Type 1 Diabetes. Immunotherapy (2013) 5:1357–66. doi: 10.2217/imt.13.130 PMC3939044 24283846

[7] Sharp RC Abdulrahim M Naser ES Naser SA. Genetic Variations of PTPN2 and PTPN22: Role in the Pathogenesis of Type 1 Diabetes and Crohn’s Disease. Front Cell Infect Microbiol (2015) 5:95. doi: 10.3389/fcimb.2015.00095 26734582 PMC4689782

[8] Neufeld M Blizzard RM. Polyglandular Autoimmune Diseases. In: Pinchera A Doniach D Fenzi GF Baschieri L, editors. Symposium on Autoimmune Aspects of Endocrine Disorders. New York: Academic Press (1980). p. 357–65.

[9] Nishihara E Amino N Kudo T Ito M Fukata S Nishikawa M. Comparison of Thyroglobulin and Thyroid Peroxidase Antibodies Measured by Five Different Kits in Autoimmune Thyroid Diseases. Endocr J (2017) 64:955–61. doi: 10.1507/endocrj 28768936

[10] Murasugi S Ito A Omori T Nakamura S Tokushige K. Clinical Characterization of Ulcerative Colitis in Patients With Primary Sclerosing Cholangitis. Gastroenterol Res Pract (2020) 2020:7969628. doi: 10.1155/2020/7969628 33224192 PMC7669346

[11] Jairath V Feagan BG. Global Burden of Inflammatory Bowel Disease. Lancet Gastroenterol Hepatol (2020) 5:2–3. doi: 10.1016/S2468-1253(19)30358-9 31648974

[12] Jostins L Ripke S Weersma RK Duerr RH McGovern DP Hui KY. Host-Microbe Interactions Have Shaped the Genetic Architecture of Inflammatory Bowel Disease. Nature (2012) 491:119–24. doi: 10.1038/nature11582 PMC3491803 23128233

[13] Lu S Gong J Tan Y Liu D. Epidemiologic Association Between Inflammatory Bowel Diseases and Type 1 Diabetes Mellitus: A Meta-Analysis. J Gastrointestin Liver Dis (2020) 29:407–13. doi: 10.15403/jgld-798 32919423

[14] Fu Y Lee CH Chi CC. Association of Psoriasis With Inflammatory Bowel Disease: A Systematic Review and Meta-Analysis. JAMA Dermatol (2018) 154:1417–23. doi: 10.1001/jamadermatol.2018.3631 PMC6583370 30422277

[15] Kang EA Han K Chun J Soh H Park S Im JP. Increased Risk of Diabetes in Inflammatory Bowel Disease Patients: A Nationwide Population-Based Study in Korea. J Clin Med (2019) 8:343. doi: 10.3390/jcm8030343 PMC6463263 30862129

[16] Hashimoto K Maruyama H Nishiyama M Asaba K Ikeda Y Takao T. Susceptibility Alleles and Haplotypes of Human Leukocyte Antigen DRB1, DQA1, and DQB1 in Autoimmune Polyglandular Syndrome Type III in Japanese Population. Horm Res (2005) 64:253–60. doi: 10.1159/000089293 16254435

